# First long‐term trajectory of an ocean sunfish ( *Mola mola* L.) from the northwestern Mediterranean

**DOI:** 10.1111/jfb.15831

**Published:** 2024-06-03

**Authors:** Tristan Rouyer, A. Landreau, O. Derridj, S. Bonhommeau, R. Frejafond, B. Wendling, V. Kerzerho

**Affiliations:** ^1^ MARBEC, University of Montpellier, CNRS, IFREMER, IRD Sète France; ^2^ IFREMER DOI Rue Jean Bertho France; ^3^ FREJAFOND Sète France; ^4^ SATHOAN Sète France; ^5^ LIRMM, University of Montpellier, CNRS Montpellier France

**Keywords:** bycatch, electronic tagging, Mediterranean, ocean sunfish (*Mola mola* L.)

## Abstract

The ocean sunfish is a large fish for which many aspects of its ecology and biology are still poorly known. Electronic tagging was used to provide the first information on the movements of an ocean sunfish from the northwestern Mediterranean. The sunfish moved towards the Gibraltar strait over the year and displayed substantial movements in the water column. The potential of the tagging technique employed for studying its behavior and long‐term migratory dynamics, and assessing the post‐release survival of ocean sunfish is highlighted.

The ocean sunfish (*Mola mola* L.) is one of the heaviest bony fish, with a unique body shape, able to perform large‐scale movements and yet for which population structure, status, and many aspects of its biology remain poorly known (Chang et al., 2021; Hays et al., 2020; Nyegaard et al., 2020; Phillips et al., 2023; Thys et al., 2015). The *Mola mola* species occupies an extensive area of distribution and is common throughout the Mediterranean (Sousa et al., 2020).

In the Mediterranean Sea, the ocean sunfish is a bycatch of some fisheries, such as the swordfish fishery (Garibaldi, 2015), driftnets (Tudela et al., 2005), bluefin tuna (BFT) traps (Addis et al., 2013), and other large pelagics fish fisheries (Nyegaard et al., 2020). In the Gulf of Lion, located in the northwestern Mediterranean, the ocean sunfish represents a small amount of bycatch in the BFT longline fishery; preliminary information from fishermen report about one individual caught every 10 sets (source OP SATHOAN‐ECHOSEA, B. Wendling, N. Cosnard, C. Segorb, pers. comm). In the Gulf of Lion, the planktonic bloom is associated with a large quantity of jellyfish, which is an important feeding source for ocean sunfish, but far from being exclusive, that is also shared by BFT (Günther et al., 2021; Phillips et al., 2020; Thys et al., 2015). The impact of bycatch on the ocean sunfish Mediterranean population is unknown (Nyegaard et al., 2020).

Pop‐up satellite archival tags (PSATs) are tools that have allowed scientists to shed light on the post‐release survival and movement of large pelagics since the late 1990s (Block et al., 2011; Hussey et al., 2015). PSATs are electronic devices attached to marine animals that typically collect temperature, pressure, and light measurements, from which the geographic position of animals can be estimated (Nielsen et al., 2006). Electronic tagging has been used by several studies to track ocean sunfish, documenting movements of individuals from various places in the world (Chang et al., 2021; Dewar et al., 2010; Sousa et al., 2020; Thys et al., 2016), such as the Atlantic basin (Potter et al., 2011; Sims, Queiroz, Humphries, et al., 2009), but from our knowledge this species has not been tagged yet with PSATs in the Mediterranean even though individuals tagged in the Atlantic have been seen to move into the Mediterranean (Sousa et al., 2016).

In agreement with recommendations from the IUCN review panel, to improve our knowledge on the ecology of this bycatch species and as a pilot study on the post‐release survival of this species, an electronic tag was deployed on a common ocean sunfish caught as a bycatch of the French BFT longline fishery operating in the Gulf of Lion (Pillips et al., 2023). The attachment technique used is detailed, and the horizontal and vertical movements of the animal are presented and discussed in light of the literature.

The tagging was conducted onboard the longliner *Trois Frères II* (ST916523) on April 14, 2022, a vessel that targets BFT in the Gulf of Lions. The tags were all rigged with the objective to tag the juvenile BFT that are usually caught during these sets and for logistical reasons, it was decided to use the same rigging for sunfish. A 75‐cm sunfish was caught very early in the set and a protocol similar to the one used for tagging BFT was used (Rouyer et al., 2019). The sunfish was hauled aboard by hand, grabbing the animal by the base of the fins. No in‐depth investigation could be carried out, but the absence of rectangular scales detected on the body and the relatively cold waters (14°C) identified the individual as *Mola mola* (Linnaeus 1758) and not *Mola alexandrini* (Ranzani, 1839) (Sawai et al., 2018). The fish was then placed onto a thick mattress and its eyes were covered with a wet cloth. A hose was inserted in the mouth of the fish to provide a continuous supply of seawater. The fish was then measured and tagged. The fish was actively moving its fins when it was hauled out, but this stopped as soon as its eyes were covered and it remained generally calm during the rest of the operation. The individual displayed a very active swimming activity when released into the water.

The tagging technique used was as similar as possible to the technique used on BFT and that yields satisfying results, with regular long‐term retention times (Rouyer et al., 2022). The tag (Wildlife Computers, miniPAT) was rigged using a 6‐cm long monofilament and an XL Domeier anchor. To reduce the overall movement of the tag, which can be a main cause of premature release, a second anchor was used to secure the tag alongside the body of the fish, as commonly used when tagging BFT. The tag was deployed to remain parallel to the main axis of the fish, about 10 cm below the dorsal fin just above the upper lateral ridge. The anchors were inserted directly through the hard skin after creating a pre‐hole using a knife blade. The deployment technique minimized the tag's potential movements by tightly fitting it against the body of the animal. The overall tagging operation and time on deck lasted <2 min. The care and use of the animals complied with EU animal welfare laws, guidelines, and policies.

The tag was programmed to release after 365 days. The data retrieved through ARGOS transmission was analyzed using the GPE3 algorithm provided by Wildlife Computers to estimate the geolocation. Several priors (3, 4, and 6 km/h) were used to test the robustness of the reconstruction. Lower values could not be used as the model would not be able to find a solution, whereas values higher than 6 km/h appeared implausible based on in situ speed estimates from acoustic and GPS tracking or measurements from speed sensors (Cartamil & Lowe, 2004; Nakamura & Sato, 2014; Sims, Queiroz, Humphries, et al., 2009). A common practice is to retain as a “best track” the one obtained with the prior that provides the highest “score” through GPE3, a sort of proxy for goodness of fit (Rouyer et al., 2022). Daily minimum and maximum depths were collected as well as daily depth profiles, which were obtained by sampling pressure every 10 min over a whole day every 4 days. The diving activity and its evolution over time was investigated by rescaling these profiles so that daily values would range between 0 and 1. The daily average depth was also compared to the first‐order autocorrelation coefficient computed over the daily series, for which higher values reflect a smoother time series, which can be interpreted as more gradual changes in depth. For some days, temperature data were available in the form of a daily histogram indicating the percentage of time spent within temperature bins, which was used to help interpret the results.

The tag popped off 362 days after its deployment and a few days before the planned duration. The different speed priors did not permit identifying a maximum score as it kept increasing with increasing speed values. Looking at the estimated distances per day did not provide more help as they looked equally plausible. The tracks displayed a similar general movement across speed priors, mostly varying in the extent of their latitudinal movements south of the Gulf of Lion and south of the Balearic Islands. The 4 km/h prior was therefore arbitrarily selected to present the results, even though the two other priors did not appear implausible. Our results remain qualitatively insensitive to this choice. The fish headed south after tagging and went back towards the north after passing Majorca's latitude, in May/June, and went back to the Gulf of Lions, where it started to generally head southwest from August to October/November, displaying east–west movements (Figure 1, middle panel) that varied across speed priors: the higher the speed, the larger the movement. In December, at about Barcelona's longitude, the fish headed south and passed the Balearic Islands, and in January/February it displayed east–west movements that also changed in magnitude with speed priors. In March, it started to move westwards towards Gibraltar and the tag popped off before the strait.

**FIGURE 1:**
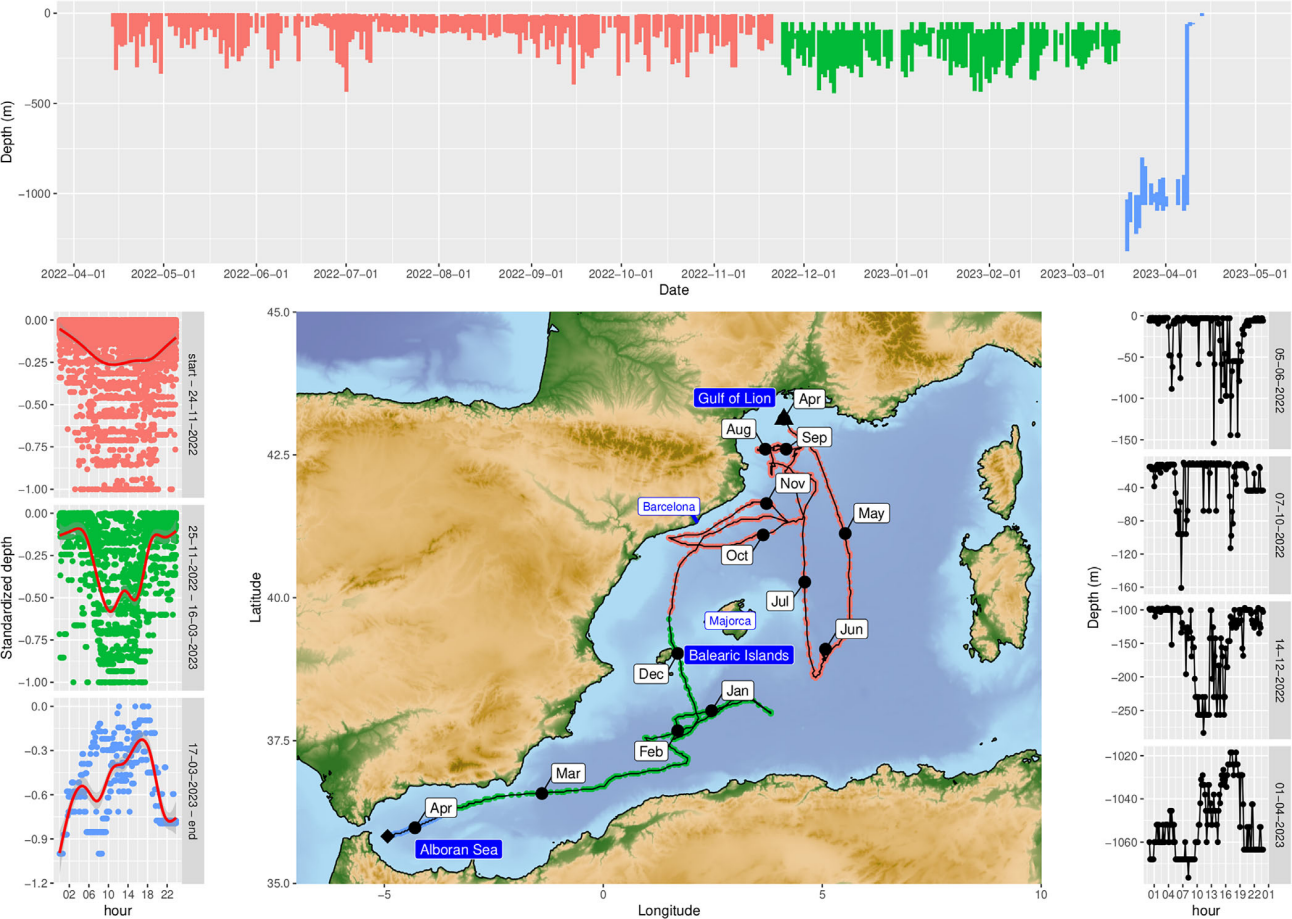
Top panel, daily minimum and maximum depth, the colors indicate periods with different diving behavior. Deployment: November 24, 2022, November 25, 2022–March 16, 2023, March 17, 2023–April 11, 2023 (pop‐off). Left panel, daily standardized depth over the day for the three different time periods. The colors correspond to the top panel and the red line is a smoother that was added to visualize the pattern. Central panel, trajectory estimated through the GPE3 algorithm for a speed prior of 4 km/h. The triangle and losange identify deployment and pop‐off (April 11, 2023) locations, respectively, whereas black points and labels identify the beginning of the different months. The colors on the trajectory identify the three different time periods. Right panel, sample daily depth profiles that illustrate the evolution of daily diving pattern over time for June 5, 2022, October 7, 2022, December 14, 2022, and April 1, 2023 (from top to bottom).

Assuming a prior of 4 km/h, as exemplified in Figure 1, the fish traveled 3956 km with a median of 8.32 km per day, covering a distance between 1.7 and 29.3 km per day 90% of the days, reaching a maximum of 59.7 km in a day in January 2023. The individual did substantial dives throughout the year, with deeper dives around mid‐day (Figure 1, top and left panels). From April to November 24, 2022, the average daily minimum and maximum depths were 7 and 153 m, respectively, with a maximum of 435 m. Until March 16, 2023, the average daily minimum and maximum depths reached were 79 and 272 m, respectively, with a maximum of 443 m. From March 17, 2023, in the Alboran Sea, until April 8, 2023 substantially deeper depths were recorded, between 890 and 1112 m on average, reaching a maximum of 1319 m. Then the average daily minimum and maximum depths became suddenly shallower at 8 and 42 m, respectively, with a maximum of 72 m. The pop‐off was recorded at 34 m deep.

The standardized daily profiles indicated that dives mostly occurred during daytime, with deeper dives more commonly found around dawn and dusk (Figure 1, right panel). This pattern seemed to become more contrasted over time with more pronounced dives at greater depths during daytime, before disappearing in late March when the fish started to spend most of its time at depths >750 m (Figure 1, left and right panels). The smoothness of the changes in depth quantified by the first‐order autocorrelation over the daily dive profiles showed a significant relationship with the median daily depth (*t*‐test, p < 0.001), meaning that the fish displayed progressive changes in depth during the day when at greater depths whereas changes in depth were more sudden at shallower depths.

Attachment techniques on animals have a key impact on tag retention times, which critically affects the ability of scientists to understand the spatio‐temporal dynamics of such species (Phillips et al., 2023). Short retention times do not permit the full extent of the spatio‐temporal variability that could be expected over a year to be captured. Achieving long‐term retention often requires trials of different techniques. In the case of ocean sunfish, the retention time of electronic tags found in the literature rarely exceeded 6 months (Chang et al., 2021; Dewar et al., 2010; Potter & Howell, 2011; Potter et al., 2011; Sims, Queiroz, Doyle, et al., 2009, Sims, Queiroz, Humphries, et al., 2009; Sousa et al., 2016; Thys et al., 2016). Retention times have been limited by the tag programming in some instances, but they also often appeared due to a premature release of the tags. Using a deployment technique similar to the one used for BFT, the retention time of 12 months achieved in this study was, as far as we know, longer than those that have currently been reported in the literature. A main aspect of our deployment technique is that the tag is tightly fitted against the body of the animal, using a double anchorage, therefore minimizing any movements. It has to be noted that the reasonable size (75 cm) of the individual tagged in the present study allowed for easy deck handling to use a double anchorage, compared to larger individuals whose size and weight can become a limitation for this kind of technique.

The year of data acquired yielded new insights into the ecology of Mediterranean ocean sunfish and a first year‐long trajectory in the northwestern Mediterranean, even though more tagging effort is needed to capture the full extent of the spatio‐temporal variability of this species' movements. It has to be noted that the uncertainty of trajectories obtained from light‐based geolocations can be substantial when no light curves can be acquired, for instance when the fish has spent a prolonged period too deep (Andrzejaczek et al., 2021). This was the case when the ocean sunfish was in the Alboran sea, but the uncertainty associated with the trajectory in that area did not appear to be larger than in others (not shown). The movement of the fish throughout the year did not indicate any yearly cycle, seasonal pattern or clear residency within a specific area of the northwestern Mediterranean (Figure 1, middle panel). It rather indicated a general movement towards Gibraltar and the Atlantic ocean as the tag popped off in front of the strait after a general movement towards the southwest. This movement towards Gibraltar was even clearer and more directional between February and the pop‐off date in April, just in front of the strait. Ocean sunfish have already been reported to transition between the Atlantic and the Mediterranean (Sousa et al., 2016). Trajectories without any clear seasonal pattern have already been observed for ocean sunfish tagged in the Pacific and in the northeast Atlantic and are very different from what can be observed on other large pelagics, such as BFT, which can display clearer alternate periods of migrations and foraging that can be identified on the tracks (Chang et al., 2021; Rouyer et al., 2022; Sousa et al., 2016). Analyzing the track with an approach allowing for switching between alternative behavioral states might yield more insight into these aspects (Nyegaard et al., 2023).

The individual tagged, despite its relatively small size, displayed substantial movements in the water column, which changed over time. The fish went progressively deeper, with deeper dives during daytime compared to nighttime. Noticeably, from mid‐March until the end of the deployment, when the fish was in the Alboran sea, it remained at depths about 1000 m, with deeper dives reaching a maximum of 1319 m. Other studies have reported substantial dives of ocean sunfish over 800 m (Potter & Howell, 2011) and over 1000 m for the close relative, giant sunfish *Mola alexandrini* (Thys et al., 2017), however, our study seems to have, so far, recorded the deepest dive for any molid species. This very unusual behavior for an ocean sunfish could be explained by a faulty depth sensor, a possibility confirmed by the manufacturer, that suggested an offset between 7 and 100 m from June onwards. Such an offset explains why the fish did not visit the surface anymore and is also consistent with the pop‐off recorded at 34 m deep, but it does not explain the sudden and prolonged deep‐dive event. The temperatures recorded during that event remained between 12 and 14°C, which is consistent with temperature values for deep water masses in the Alboran sea (Ercilla et al., 2016). A possible interpretation is that this behavior was caused by the death of the fish, maybe following a predator attack. The magnitude of the vertical movements recorded appear incompatible with a fish sinking to the bottom (Thys et al., 2015), but it may have been moved up and down the water column by deep currents (Ercilla et al., 2016), in line with the more gradual changes in depth observed during that period. It remains difficult to establish whether underwater currents can be the cause of such substantial vertical movements, which prevented the tag from detecting mortality through constant pressure. Then, as the flesh decayed, the tag eventually could have detached from the fish and rapidly surfaced.

The post‐release survival of ocean sunfish for different fisheries is largely unknown, but is of interest given its presence in the species composition of the bycatch for many fisheries (Phillips et al., 2023). Our results provide preliminary evidence that ocean sunfish can survive a catch from a longline when handled with care. The tagged individual was in good health and no injury could be noticed on it, which is likely to increase survival compared to the injured individuals that can be observed in other fisheries (Cartamil & Lowe, 2004). It goes without saying that a larger sample size, without selecting individuals based on their apparent health or absence of injury, would be required to investigate further post‐release survival and potentially generalize this result (Thys et al., 2015).

## AUTHOR CONTRIBUTIONS

T. Rouyer: designed the research and did the fieldwork, made the analyses and wrote the paper. A. Landreau made the analyses and wrote the paper. O. Derridj did the fieldwork. S. Bonhommeau designed the research and wrote the paper. R. Fréjafond did the fieldwork. B. Wendling wrote the paper. V. Kerzerho designed the research and wrote the paper.
